# No associations exists between red blood cell distribution width and serum uric acid in both sexes

**DOI:** 10.1097/MD.0000000000012707

**Published:** 2018-10-05

**Authors:** Chunmei Zhang, Zhaowei Meng, Xue Li, Ming Liu, Xiaojun Ren, Mei Zhu, Qing He, Qing Zhang, Kun Song, Qiyu Jia, Qian Chen

**Affiliations:** a Department of Nuclear Medicine; b Department of Endocrinology and Metabolism; c Department of Health Management, Tianjin Medical University General Hospital, Tianjin; d Department of Biotechnology, College of Life Science, Nanjing University, Nanjing, Jiangsu, Province, China.

**Keywords:** red blood cell distribution width, uric acid, gender, age

## Abstract

The aim of this study was to determine whether there was a significant association between red blood cell distribution width (RDW) and uric acid (UA) in a large Chinese population.

This was a cross-sectional study with an enrollment of 80,298 ostensibly healthy participants (48,971 males, 31,327 females) during the period from 2011 to 2015. In the study, database was grouped by sex and the association between RDW and UA was analyzed by quartiles of RDW.

UA values between different sexes and RDW subgroups were analyzed by 2-way analysis of variance and Bonferroni *t* tests. Prevalence of hyperuricemia in different sexes was calculated. The relationship between risks of hyperuricemia and RDW level was analyzed by binary logistic regression with or without adjustment for age and body mass index.

UA values were not all the same between different sexes and RDW subgroups. Males had significantly higher hyperuricemia prevalence than females (20.00% vs 6.48%, *P* < .01). In addition, hyperuricemia prevalence in males decreased slightly across RDW quartiles, but was stable in females. No significant association between hyperuricemia risk and RDW was found in both sexes according to the results of multivariate logistic regression analysis. Similarly, negative results were also observed in multivariate linear analysis when both RDW and UA were considered as continuous variable.

We could not find any significant relationship between RDW and UA in both sexes.

## 1 Introduction

Red blood cell distribution width (RDW) is an erythrocyte parameter, which is calculated by dividing the standard deviation of erythrocyte volume with erythrocyte mean corpuscular volume (MCV) and then multiplying by 100. RDW has traditionally been used for the differential diagnosis of anemia.^[1]^ In recent years, several studies have demonstrated that increased RDW is associated with a poor prognosis in clinical settings of cardiovascular and thrombotic diseases,^[2]^ stroke,^[3]^ coronary artery disease,^[4]^ diabetes mellitus,^[5]^ hypertension,^[6]^ metabolic syndrome (MS),^[7,8]^ renal function damage,^[5,9]^ and it can be a predictor of all-cause mortality in general population.^[2]^ However, it has not been well ascertained whether anisocytosis might be a cause or a simple epiphenomenon of the underlining conditions of the above-mentioned diseases, such as inflammation, oxidative stress, under nutrition, and so forth or maybe an element of both.^[2]^

Uric acid (UA) is the final metabolism product of endogenous and exogenous purine nucleotide.^[10,11]^ Approximately 70% of UA is excreted in the kidney while the remaining part is excreted by the intestinal tract.^[12]^ Higher concentration of UA leads to development of hyperuricemia and gout. Elevated serum UA is also suggested to be associated with type 2 diabetes,^[13]^ hypertension,^[14]^ cardiovascular diseases (CVD),^[15,16]^ thyroid dysfunction,^[17]^ and MS.^[18]^

From above description, we can see that both RDW and UA have correlations with multiple diseases, so we want to explore whether a correlation exists between RDW and UA that seems irrelevant. There was only 1 publication we could retrieve which focused on the relationship between RDW and UA. Luo et al^[19]^ found that RDW was independently correlated with UA after adjustments of several related factors in 512 patients with newly diagnosed hypertension without treatment. This earlier research had limitations in several aspects, such as small sample size, recruiting only patients with hypertension, and so forth. In fact, taken into account of the above-mentioned characteristics of RDW and UA, we want to explore whether a relationship between RDW and UA exists in healthy people rather than in people who suffer from any particular diseases, emphasizing on different genders with large sample size.

Therefore, the goal of present study was to systematically evaluate the association between RDW and UA in a large number of Chinese individuals. Special attention was paid to the gender differences.

## 2 Materials and methods

### 2.1 Design

This study was based on a cross-sectional, community-based health check investigation, which was initiated nearly 5 years ago, and conducted in Tianjin Medical University General Hospital. The Departments of Health Management, Endocrinology and Metabolism, and Nuclear Medicine collaborated in this research. The protocol was developed and executed as previously by our group in accordance with the Declaration of Helsinki.^[17,18,20–25]^ For the current analysis, all subjects were self-reported as healthy without any known previous diseases. All participants were asked to complete a questionnaire about medical history, lifestyle, and alcohol intake and then provide an overnight fasting blood sample. All participants were healthy as they reported. To avoid the influence of confounding factors, the exclusion criteria were subjects with disease history of blood disease; subjects with any diseases or taking any medicine that might affect UA; subjects with a history of cardiac diseases, kidney diseases, and liver diseases. All participates were required to receive a questionnaire inquiries. During the period from 2011 to 2015, a total of 80,298 candidates (48,971 males, 31,327 females) with complete data for analysis were finally included in this particular subject.

### 2.2 Ethics

The institutional review board and ethic committee of Tianjin Medical University General Hospital approved the ethical, methodological, and protocol aspects of this investigation. We confirm that all methods in the current study were carried out in accordance with the relevant guidelines and regulations.

All participants in this research provided their informed consents, so every people participated in our study voluntarily and comprehended all aspects about the research.

### 2.3 Measurements

Anthropometric examinations and fasting blood sample tests of participants were performed when participants visited our institution. Height, waist, and weight were measured in centimeters and kilograms, respectively. Body mass index (BMI) was calculated by dividing body weight (kg) by the square of body height (m^2^). Blood pressure was measured with a standard mercury sphygmomanometer after a sedentary for at least 5 minutes. Alanine aminotransferase (ALT), total bilirubin, blood urea nitrogen, creatinine, UA, total cholesterol, triglycerides (TG), high-density lipoprotein, low-density lipoprotein (LDL), and glucose were determined enzymatically by an auto-analyzer (Hitachi Corporation, Tokyo, Japan). C-reactive protein (CRP) was done on an analyzer (Hebei Diagnostics, Shijiazhuang, China), and erythrocyte sedimentation rate (ESR) was determined by Wintergreen's method (Yakun Diagnostics, Tianjin, China). White blood cell (WBC), granulocyte, lymphocyte, red blood cell (RBC), hemoglobin, RDW, RBC specific volume, MCV, mean corpuscular hemoglobin, mean corpuscular hemoglobin concentration, platelet, platelet distribution width, and mean platelet volume were measured with a hemocytometer analyzer (Sysmex Corporation, Kobe, Japan).

### 2.4 Definition

The diagnosis of hyperuricemia required a UA level of >420 μmol/L for males and >360 μmol/L for females.^[17,18]^ The grouping method for RDW was based on quartiles of the measurements.

### 2.5 Statistical analysis

In order to see if there exit any differences in different intervals, RDW was grouped based on quartiles rather than continuous variable. UA values were presented as mean ± standard deviation, 2-way analysis of variance (ANOVA) and Bonferroni post hoc test were accomplished to analyze differences of UA between different RDW quartiles and sexes. Chi-squared test was applied to compare hyperuricemia prevalence differences between subgroups. The binary logistic regression models were made to calculate the crude odd ratio (OR) for hyperuricemia with 95% confidence intervals by stratifying data with RDW quartiles. In the last, the correlation between UA and RDW was explored by univariate and multiple linear regression analyses. Backward method was used for variable selection in the multiple linear regression analyses. Statistical analysis was conducted by the Statistical Package for Social Sciences (SPSS version 17.0, Chicago, IL) and *P* < .05 was regarded as significance.

## 3 Results

### 3.1 UA values between different sexes and RDW subgroups

In Table 1, 2-way ANOVA results showed the statistics for sex was F = 37,472.017 (*P* < .01), and for RDW subgroups was F = 17.525 (*P* < .01). For interaction of sex × RDW subgroups was F = 6.240 (*P* < .01). Further Bonferroni post hoc test demonstrated the significant differences in UA between subgroups of RDW ≤ 12.2 and 12.2 < RDW ≤ 12.6, 12.6 < RDW ≤ 13.0, RDW > 13.0, and the differences between 12.6 < RDW ≤ 13.0 and RDW > 13.0 subgroups (*P* < .05).

**Table 1:**
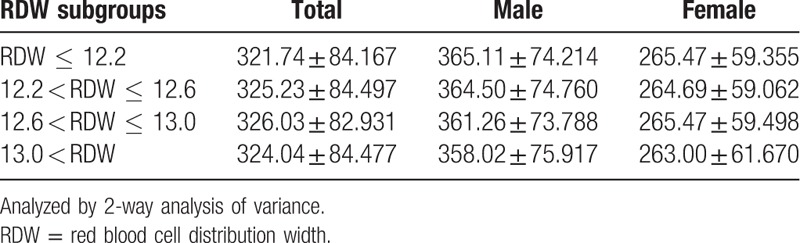
Uric acid values between different sexes and red blood cell distribution width subgroups.

### 3.2 Prevalence of hyperuricemia in different genders

The prevalence of hyperuricemia in this population was 14.76% (11,855/80,298). Males had significantly higher hyperuricemia prevalence than females (Table 2). All RDW quartiles demonstrated the same pattern of prevalence differences in opposite sex. In addition, the prevalence of hyperuricemia decreased to a certain degree across the RDW quartiles in males and remained roughly stable in females.

**Table 2:**
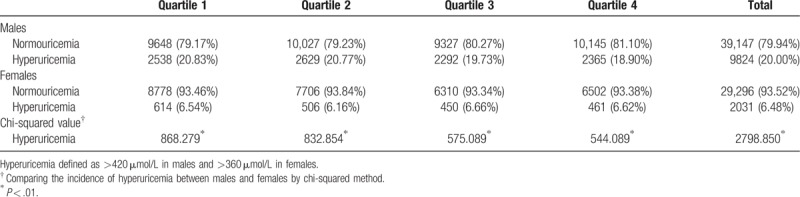
Incidence (and case number count) in different red blood cell distribution width quartiles and genders.

### 3.3 Risks of hyperuricemia in different RDW quartiles and other factors

In our study, we used binary logistic regression models to analyze the risks of developing hyperuricemia in relation to different variables. OR calculation was performed with highest quartile of RDW as reference (Table 3), and other risk factors included age and BMI were adjusted. We could not identify significant risks after adjustment with covariates for hyperuricemia in both males and females, suggesting covariates greatly influenced the relationship between RDW and UA.

**Table 3:**
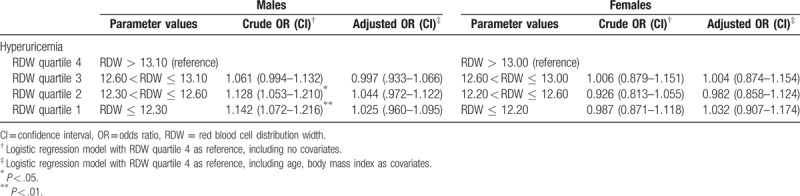
The risks of hyperuricemia according to red blood cell distribution width quartiles in different genders.

From Table 4, we concluded that BMI, ALT, and TG display detrimental effects to develop hyperuricemia in both sexes while the effect of age in the opposite. When RDW is viewed as continuous variable, the relations of RDW and CRP to hyperuricemia were consistent with the previous results.

**Table 4:**
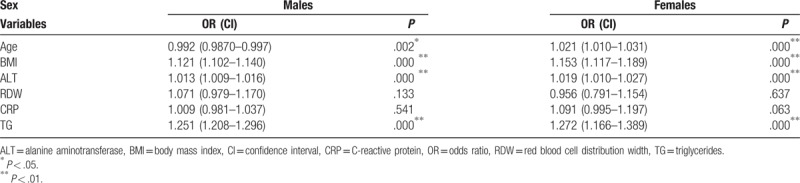
The likelihood of developing hyperuricemia in different variables.

### 3.4 Relationship between UA and RDW by univariate and multiple linear analyses

Univariate linear analyses were used first to demonstrate any association between UA versus RDW, age, BMI, and all other factors (Table 5). We found that the correlation between UA and RDW existed in male but not in female, besides, we did not find significant relationship between UA and ESR in both genders. Multiple linear regression models were further performed, and the variables which were significant in the univariate analysis were considered as covariates. The insignificance of RDW retained according to the result of multiple linear regression. In general, no relationship between UA and RDW could be rendered in both males and females (Table 6).

**Table 5:**
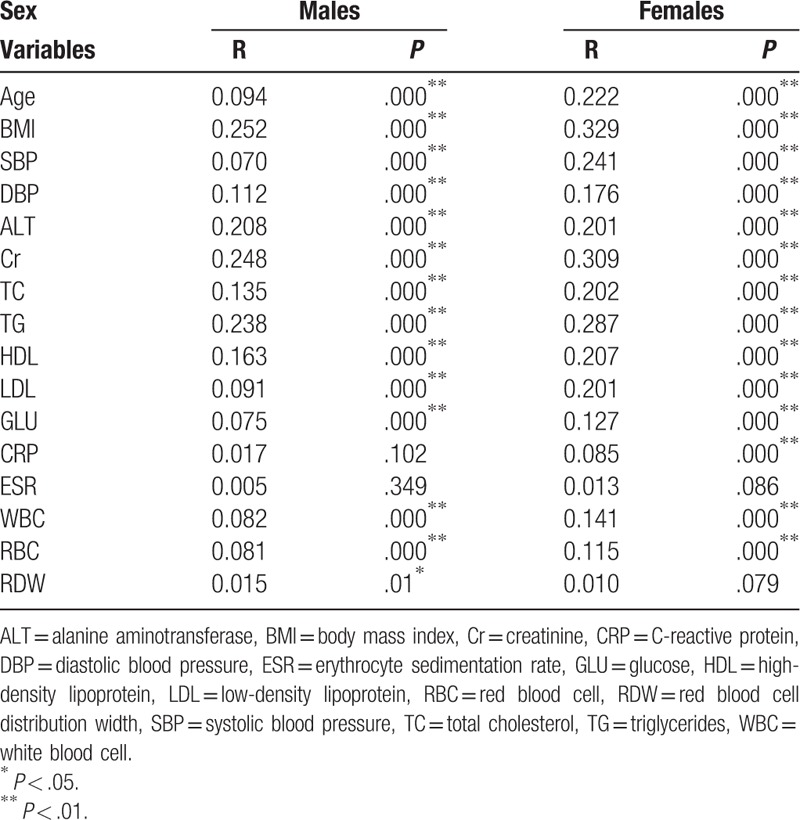
Relationship between uric acid and different variables by univariate linear analyses.

**Table 6:**
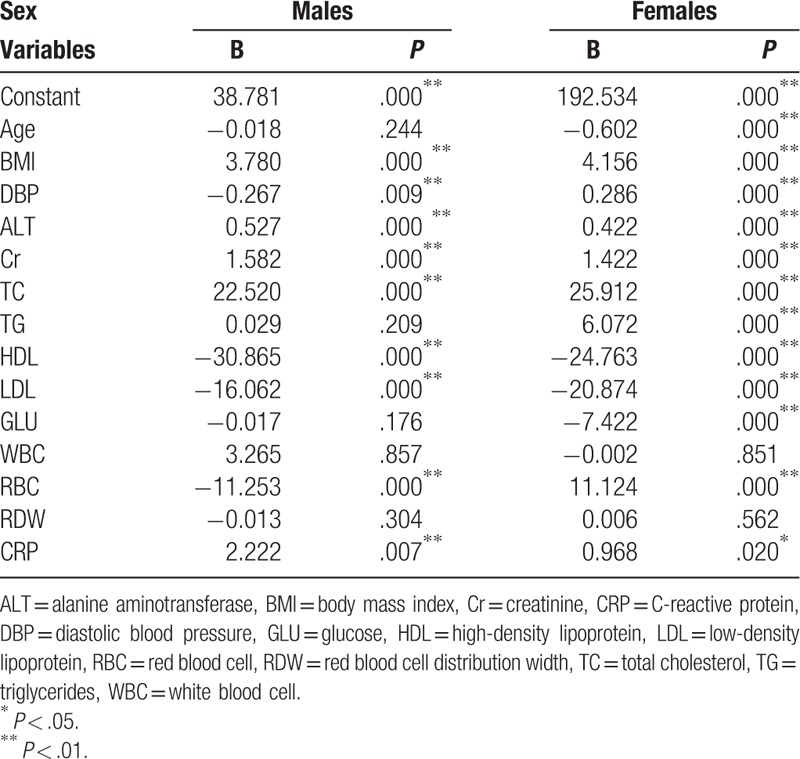
Relationship between uric acid and red blood cell distribution width by multiple linear analyses.

## 4 Discussion

The current investigation demonstrates that in healthy population, males have significantly higher hyperuricemia prevalence than females. Hyperuricemia prevalence in males decreases slightly across RDW quartiles, but was stable in females. More importantly, this is the first study that demonstrated no association existed between RDW and UA in healthy people without any diseases in either gender, indicating that covariates could greatly confound the relationship between RDW and UA.

RDW is a parameter derived from routine blood cell counts, which can be automatically calculated from MCV by hematology analyzers. RDW reflects the degree of heterogeneity in size of the peripheral erythrocytes. There are many factors can affect RDW value, which include iron, vitamin B12, folic acid, erythropoietin, and so forth. An increased RDW suggests a profound deregulation of erythrocyte homeostasis including both impaired erythropoiesis and abnormal erythrocyte metabolism.^[26]^ And elevated RDW is accompanied by lower erythrocyte deformability.

There are elevating numbers of researches evaluating diagnostic and prognostic values of RDW in diseases besides hematology. However, apparent contradictions exist. In CVD, for instance, a review^[2]^ demonstrated that a higher level of RDW was associated with adverse outcomes in patients with or without CVD even after adjusting with several confounding variables. The authors concluded that the states of oxidative stress and inflammation might be important determinants of RDW. As such, Marinkovic et al^[27]^ suggested that oxidative stress could shorten erythrocytes’ lifespan and make them prone to be hemolytic. Inflammatory cytokines,^[28]^ such as tumor necrosis factor α and interleukin, are strongly related to ineffective erythropoiesis by desensitizing bone marrow erythroid progenitors to erythropoietin, inhibiting RBC maturation, and thereby promoting anisocytosis. Nevertheless, in another study,^[29]^ no association was found between RDW concentrations and all-cause mortality in non-ST elevation myocardial infarction patients although a positive association between high levels of RDW and the severity of coronary artery disease was shown. And the relationship between RDW and CRP was yet to be clarified. Likewise, another investigation conducted by Yoon et al^[30]^ elucidated that baseline RDW was not associated with adverse outcomes in 337 CVD patients with end-stage renal diseases treated by dialysis. It did not clearly demonstrate whether elevated serum RDW might be a risk factor or only an epiphenomenon.

For another example, in the case of RDW and hypertension, incompatible studies were also presented. In a research,^[6]^ significant correlation was shown between increased RDW and blood pressure. RDW was higher in patients with prehypertension and hypertension than healthy controls independent of age, inflammatory status, and anemia, which could be caused by the neuroendocrine system activation. In the meantime, association between ESR, WBC count, and RDW had not been found in this study although inflammation was suggested to be a risk factor for hypertension.^[31]^ On the contrary, an investigation enrolling 9808 participants found that subjects with hypertension had significant lower RDW compared with controls.^[32]^ This study proposed that gene polymorphisms and deletions might assume the main responsibility in regulating hypertension,^[33]^ which indicated that RDW might only be a by standing factor.

There was only 1 previous investigation,^[19]^ studied the relationship between RDW and UA, and the results in this study were generally different from our research. We considered that variations existed because of the followings points. First, only 512 middle-aged hypertensive participants without antihypertensive treatment were recruited in the previous study, but the present study focused on the relationship between RDW and UA with the largest sample size until now, and our participants were from a general health check program. Second, the definition for high UA was >334.9 μmol/L in the former without gender stratification, yet we defined hyperuricemia as UA >420 μmol/L for males and >360 μmol/L for females, respectively. Third, the former study did not analyze the data in different genders. However, all analyses in our investigation were conducted in men and women separately.

There could be some postulations to explain these contradictory results. On the one hand, we believe that several crucial factors affecting UA may have a great impact on the results, including lifestyle, oxidative status, and genetic background. First, high intake of fructose-rich industrialized food,^[34]^ alcohol intake,^[35]^ and reduced renal excretion^[12]^ can raise UA concentration. Adiposity, weight gain, hypertension, and diuretics have been considered as risk factors for gout in men, while weight loss is protective.^[36]^ Second, Hamur et al^[37]^ suggested that elevation in serum UA level contributed to oxidative stress, endothelial dysfunction, and smooth muscle cell proliferation. UA functions as an antioxidant in the presence of native LDL taken from human plasma, but in response to mildly oxidized LDL, UA becomes a pro-oxidant. UA also plays a pro-oxidant role in the process of adverse outcomes.^[38]^ Third, genetic factors are considered important for gout development. In fact, homozygous deleterious mutation and polymorphisms in the SLC22A12 gene was suggested to be related with a high level of UA and gout in a study.^[39]^ Another study elucidated that C677 mutation in methylene tetrahydrofolate reductase gene was a risk factor of hyperuricemia in elderly Korean men.^[40]^ Among different genotypes (GG, GT, and TT), different UA levels were shown.^[41]^ On the other hand, there are also many influential factors for RDW (e.g., measuring technique, age, gender), which should be considered when interpreting the results. A study conducted by Gunawardena et al^[42]^ elucidated that RDW value could changed significantly from 24 hours of storage at different temperatures. Lippi et al^[43]^ demonstrated that different analyzers showed broad variation of RDW values in the same blood samples. But Hoffmann et al^[44]^ thought that measuring instruments did not change RDW levels significantly. Furthermore, inconsistent results were shown with respect to age and gender. It has been suggested that RDW values increased across age groups and was slightly but significantly higher in females than in males.^[45]^

The present study has several shortcomings. First, the relationship cannot be confirmed because of the nature of this cross-sectional study. Further prospective and interventional studies are necessary to explain the question. Second, inflammatory cytokines (such as interleukin and tumor necrosis factor α) were not measured in this investigation due to budget shortage. Third, we checked blood parameters only on a single blood sampling, but we did not double check due to budget shortage. This may be less precise than repeated measurements. And finally, detailed food recall and some other consumed drugs, which could influence hematological parameters or metabolism, should be recorded in specific details for risk stratification in further research.

In conclusion, we found no significant relationship between RDW and UA in both sexes in the current study. There are many factors that affect RDW and UA, so the changes of RDW and UA might only be an epiphenomenon. There was little research for the relationship between RDW and UA and we can only make a conclusion that no relationships exist between RDW and UA in this study, we want to exanimate the cofounders and mediators for the relationship in future study.

## Acknowledgments

The authors thank Professor Yaguang Fan (a dedicated statistician from Tianjin Key Laboratory of Lung Cancer Metastasis and Tumor Microenvironment, Tianjin Lung Cancer Institute, Tianjin Medical University General Hospital, Tianjin, China) for reviewing statistical analyses of the paper.

## Author contributions

**Conceptualization:** Zhaowei Meng, Ming Liu, Qing Zhang.

**Data curation:** Xiaojun Ren, Mei Zhu, Qing He, Kun Song, Qiyu Jia, Qian Chen.

**Formal analysis:** Xiaojun Ren, Mei Zhu, Qing He, Kun Song, Qiyu Jia, Qian Chen.

**Funding acquisition:** Zhaowei Meng, Ming Liu, Qing Zhang.

**Investigation:** Xiaojun Ren, Mei Zhu, Qing He, Kun Song, Qiyu Jia, Qian Chen.

**Methodology:** Xiaojun Ren, Mei Zhu, Qing He, Kun Song, Qiyu Jia, Qian Chen.

**Writing – original draft:** Chunmei Zhang, Xue Li.

**Writing – review & editing:** Zhaowei Meng, Ming Liu, Qing Zhang.

## References

[1] Evans TC Jehle D The red blood cell distribution width. J Emerg Med 1991;9 (suppl 1):71–4. 1955687 10.1016/0736-4679(91)90592-4

[2] Montagnana M Cervellin G Meschi T The role of red blood cell distribution width in cardiovascular and thrombotic disorders. Clin Chem Lab Med 2011;50:635–41. 22505527 10.1515/cclm.2011.831

[3] Kaya A Isik T Kaya Y Relationship between red cell distribution width and stroke in patients with stable chronic heart failure: a propensity score matching analysis. Clin Appl Thromb Hemost 2015;21:160–5. 23804231 10.1177/1076029613493658

[4] Bujak K Wasilewski J Osadnik T The prognostic role of red blood cell distribution width in coronary artery disease: a review of the pathophysiology. Dis Markers 2015;2015:824624. 26379362 10.1155/2015/824624 PMC4563066

[5] Zhang M Zhang Y Li C Association between red blood cell distribution and renal function in patients with untreated type 2 diabetes mellitus. Ren Fail 2015;37:659–63. 25682974 10.3109/0886022X.2015.1010938

[6] Tanindi A Topal FE Topal F Red cell distribution width in patients with prehypertension and hypertension. Blood Press 2012;21:177–81. 22243409 10.3109/08037051.2012.645335

[7] Sanchez-Chaparro MA Calvo-Bonacho E Gonzalez-Quintela A Higher red blood cell distribution width is associated with the metabolic syndrome: results of the Ibermutuamur CArdiovascular RIsk assessment study. Diabetes Care 2010;33:e40. 20190288 10.2337/dc09-1707

[8] Laufer Perl M Havakuk O Finkelstein A High red blood cell distribution width is associated with the metabolic syndrome. Clin Hemorheol Microcirc 2015;63:35–43. 26444609 10.3233/CH-151978

[9] Li ZZ Chen L Yuan H Relationship between red blood cell distribution width and early-stage renal function damage in patients with essential hypertension. J Hypertens 2014;32:2450–5. 25232756 10.1097/HJH.0000000000000356

[10] Richette P Bardin T Gout. Lancet 2010;375:318–28. 19692116 10.1016/S0140-6736(09)60883-7

[11] Choi HK Mount DB Reginato AM Pathogenesis of gout. Ann Intern Med 2005;143:499–516. 16204163 10.7326/0003-4819-143-7-200510040-00009

[12] Bellomo G Uric acid and chronic kidney disease: a time to act? World J Nephrol 2013;2:17–25. 24175261 10.5527/wjn.v2.i2.17 PMC3782226

[13] Kodama S Saito K Yachi Y Association between serum uric acid and development of type 2 diabetes. Diabetes Care 2009;32:1737–42. 19549729 10.2337/dc09-0288 PMC2732137

[14] Masuo K Kawaguchi H Mikami H Serum uric acid and plasma norepinephrine concentrations predict subsequent weight gain and blood pressure elevation. Hypertension 2003;42:474–80. 12953019 10.1161/01.HYP.0000091371.53502.D3

[15] Freedman DS Williamson DF Gunter EW Relation of serum uric acid to mortality and ischemic heart disease. The NHANES I Epidemiologic Follow-up Study. Am J Epidemiol 1995;141:637–44. 7702038 10.1093/oxfordjournals.aje.a117479

[16] Fang J Alderman MH Serum uric acid and cardiovascular mortality the NHANES I epidemiologic follow-up study, 1971–1992. National Health and Nutrition Examination Survey. Jama 2000;283:2404–10. 10815083 10.1001/jama.283.18.2404

[17] Zhang J Meng Z Zhang Q Gender impact on the correlations between subclinical thyroid dysfunction and hyperuricemia in Chinese. Clin Rheumatol 2016;35:143–9. 25875744 10.1007/s10067-015-2867-4

[18] Zhang Q Lou S Meng Z Gender and age impacts on the correlations between hyperuricemia and metabolic syndrome in Chinese. Clin Rheumatol 2011;30:777–87. 21181218 10.1007/s10067-010-1660-7

[19] Luo M Li ZZ Li YY Relationship between red cell distribution width and serum uric acid in patients with untreated essential hypertension. Sci Rep 2014;4:7291. 25464864 10.1038/srep07291 PMC4252898

[20] Zhou P Meng Z Liu M The associations between leukocyte, erythrocyte or platelet, and metabolic syndrome in different genders of Chinese. Medicine 2016;95:e5189. 27858856 10.1097/MD.0000000000005189 PMC5591104

[21] Meng Z Liu M Zhang Q Gender and age impacts on the association between thyroid function and metabolic syndrome in Chinese. Medicine 2015;94:e2193. 26683929 10.1097/MD.0000000000002193 PMC5058901

[22] Meng Z Liu M Zhang Q Gender and age impact on the association between thyroid-stimulating hormone and serum lipids. Medicine 2015;94:e2186. 26656346 10.1097/MD.0000000000002186 PMC5008491

[23] Liu L Lou S Xu K Relationship between lifestyle choices and hyperuricemia in Chinese men and women. Clin Rheumatol 2013;32:233–9. 23132661 10.1007/s10067-012-2108-z

[24] Ren X Meng Z Liu M No associations exist between mean platelet volume or platelet distribution width and thyroid function in Chinese. Medicine 2016;95:e4573. 27749526 10.1097/MD.0000000000004573 PMC5059028

[25] Wang S Zhang J Zhu L Association between liver function and metabolic syndrome in Chinese men and women. Sci Rep 2017;7:44844. 28317840 10.1038/srep44844 PMC5357848

[26] Salvagno GL Sanchis-Gomar F Picanza A Red blood cell distribution width: a simple parameter with multiple clinical applications. Crit Rev Clin Lab Sci 2015;52:86–105. 25535770 10.3109/10408363.2014.992064

[27] Marinkovic D Zhang X Yalcin S Foxo3 is required for the regulation of oxidative stress in erythropoiesis. J Clin Invest 2007;117:2133–44. 17671650 10.1172/JCI31807 PMC1934587

[28] Macdougall IC Cooper A The inflammatory response and epoetin sensitivity. Nephrol Dial Transplant 2002;17 (suppl 1):48–52. 10.1093/ndt/17.suppl_1.48 11812913

[29] Sahin O Akpek M Sarli B Association of red blood cell distribution width levels with severity of coronary artery disease in patients with non-ST elevation myocardial infarction. Med Princ Pract 2015;24:178–83. 25531370 10.1159/000369396 PMC5588291

[30] Yoon HE Kim SJ Hwang HS Progressive rise in red blood cell distribution width predicts mortality and cardiovascular events in end-stage renal disease patients. PLoS ONE 2015;10:e0126272. 25961836 10.1371/journal.pone.0126272 PMC4427112

[31] Sesso HD Buring JE Rifai N C-reactive protein and the risk of developing hypertension. Jama 2003;290:2945–51. 14665655 10.1001/jama.290.22.2945

[32] Emamian M Hasanian SM Tayefi M Association of hematocrit with blood pressure and hypertension. J Clin Lab Anal 2017;31: 10.1002/jcla.22124 PMC6816830 28105697

[33] Patel SK Velkoska E Freeman M From gene to protein-experimental and clinical studies of ACE2 in blood pressure control and arterial hypertension. Front Physiol 2014;5:227. 25009501 10.3389/fphys.2014.00227 PMC4067757

[34] Choi HK Atkinson K Karlson EW Purine-rich foods, dairy and protein intake, and the risk of gout in men. N Engl J Med 2004;350:1093–103. 15014182 10.1056/NEJMoa035700

[35] Choi HK Curhan G Soft drinks, fructose consumption, and the risk of gout in men: prospective cohort study. Bmj 2008;336:309–12. 18244959 10.1136/bmj.39449.819271.BE PMC2234536

[36] Choi HK Atkinson K Karlson EW Obesity, weight change, hypertension, diuretic use, and risk of gout in men: the health professionals follow-up study. Arch Intern Med 2005;165:742–8. 15824292 10.1001/archinte.165.7.742

[37] Hamur H Onk OA Vuruskan E Determinants of chronic total occlusion in patients with peripheral arterial occlusive disease. Angiology 2017;68:151–8. 27059289 10.1177/0003319716641827

[38] Patterson RA Horsley ET Leake DS Prooxidant and antioxidant properties of human serum ultrafiltrates toward LDL: important role of uric acid. J Lipid Res 2003;44:512–21. 12562831 10.1194/jlr.M200407-JLR200

[39] Vazquez-Mellado J Alvarado-Romano V Burgos-Vargas R Homozygous frameshift mutation in the SLC22A12 gene in a patient with primary gout and high levels of serum uric acid. J Clin Pathol 2007;60:947–8. 17660342 10.1136/jcp.2006.037473 PMC1994487

[40] Hong YS Lee MJ Kim KH The C677 mutation in methylene tetrahydrofolate reductase gene: correlation with uric acid and cardiovascular risk factors in elderly Korean men. J Korean Med Sci 2004;19:209–13. 15082892 10.3346/jkms.2004.19.2.209 PMC2822300

[41] Shima Y Teruya K Ohta H Association between intronic SNP in urate-anion exchanger gene, SLC22A12, and serum uric acid levels in Japanese. Life Sci 2006;79:2234–7. 16920156 10.1016/j.lfs.2006.07.030

[42] Gunawardena D Jayaweera S Madhubhashini G Reliability of parameters of complete blood count with different storage conditions. J Clin Lab Anal 2017;31: 10.1002/jcla.22042 PMC6816920 27565129

[43] Lippi G Pavesi F Bardi M Lack of harmonization of red blood cell distribution width (RDW). Evaluation of four hematological analyzers. Clin Biochem 2014;47:1100–3. 24925288 10.1016/j.clinbiochem.2014.06.003

[44] Hoffmann JJ Nabbe KC van den Broek NM Effect of age and gender on reference intervals of red blood cell distribution width (RDW) and mean red cell volume (MCV). Clin Chem Lab Med 2015;53:2015–9. 26536583 10.1515/cclm-2015-0155

[45] Lippi G Salvagno GL Guidi GC Red blood cell distribution width is significantly associated with aging and gender. Clin Chem Lab Med 2014;52:e197–9. 24897405 10.1515/cclm-2014-0353

